# Development of Inherently Flame—Retardant Phosphorylated PLA by Combination of Ring-Opening Polymerization and Reactive Extrusion

**DOI:** 10.3390/ma13010013

**Published:** 2019-12-18

**Authors:** Rosica Mincheva, Hazar Guemiza, Chaimaa Hidan, Sébastien Moins, Olivier Coulembier, Philippe Dubois, Fouad Laoutid

**Affiliations:** 1 Laboratory of Polymeric and Composite Materials, University of Mons, Place du Parc 23, 7000 Mons, Belgium; rosica.mincheva@umons.ac.be (R.M.); Sebastien.MOINS@umons.ac.be (S.M.); Olivier.coulembier@umons.ac.be (O.C.); philippe.dubois@umons.ac.be (P.D.); 2 Polymeric and Composite Materials Unit, Materia Nova Research Center, Nicolas Copernic 3, 7000 Mons, Belgium; hazarguemiza@gmail.com (H.G.); chai.hidan@gmail.com (C.H.)

**Keywords:** reactive flame retardancy, PLA ROP, chain extension, DOPO

## Abstract

In this study, a highly efficient flame-retardant bioplastic poly(lactide) was developed by covalently incorporating flame-retardant DOPO, that is, 9,10-dihydro-oxa-10-phosphaphenanthrene-10-oxide. To that end, a three-step strategy that combines the catalyzed ring-opening polymerization (ROP) of L,L-lactide (L,L-LA) in bulk from a pre-synthesized DOPO-diamine initiator, followed by bulk chain-coupling reaction by reactive extrusion of the so-obtained phosphorylated polylactide (PLA) oligomers (DOPO-PLA) with hexamethylene diisocyanate (HDI), is described. The flame retardancy of the phosphorylated PLA (DOPO-PLA-PU) was investigated by mass loss cone calorimetry and UL-94 tests. As compared with a commercially available PLA matrix, phosphorylated PLA shows superior flame-retardant properties, that is, (i) significant reduction of both the peak of heat release rate (pHRR) and total heat release (THR) by 35% and 36%, respectively, and (ii) V0 classification at UL-94 test. Comparisons between simple physical DOPO-diamine/PLA blends and a DOPO-PLA-PU material were also performed. The results evidenced the superior flame-retardant behavior of phosphorylated PLA obtained by a reactive pathway.

## 1. Introduction

Bio-based polymers have recently attracted a growing interest to improve the sustainability of plastics. These green materials gained an increasing demand for durable applications because they present a reduced carbon footprint compared with those produced from fossil carbon. However, their use in high-performance applications is limited because of their high flammability. In fact, fire retardancy of polymeric materials has become an important requirement prior to being used in several technical applications.

Polylactide (PLA) is one of the most promising biopolymers that is increasingly being used for technical applications [1,2], owing to its various advantages. It is produced from annually renewable resources and it presents a high stiffness, high degree of transparency in addition to its relatively low cost, and large production volume. However, PLA suffers from some shortcomings because some of its properties such as impact resistance, ductility, tensile strength, service temperature, long-term stability, and flame-retardant behavior need to be improved.

Currently, the additive way is the most common approach for enhancing PLA flame-retardant properties. It consists of the physical blending of PLA with flame-retardant (FR) additives such as organic phosphorus [3,4,5], intumescent systems [6,7], and mineral fillers [8].

Among these, the organic phosphorus flame retardants are the most adapted for PLA. The presence of phosphorous promotes the formation of crosslinked or carbonized structures that act as an insulator layer during the combustion, thus protecting the polymer underneath from the heat and limiting the volatilization of fuel [9]. The association of phosphorus-based flame-retardant additives with bio-based char forming agents such as lignin [10,11,12,13], cellulose [14,15], or tannic acid [16,17] presents an efficient way for enhancing flame retardant performances and reducing the environmental impact of the FR system. An incorporation rate of at least 20 wt.% is required prior to obtaining enhanced flame-retardant performances. However, PLA is very sensitive to thermal degradation during melt processing and the incorporation of these additives generally induces an important reduction of polymer molecular weight, significantly affecting its mechanical properties.

The integration of phosphorous directly along the PLA chain presents an interesting alternative to the additive way. Using a reactive phosphorous-based molecule as a PLA chain extension is an easy way allowing the incorporation of phosphorous in the backbone of the PLA macromolecule. With this view, Wang et al. [18] used ethyl phosphorodichloridate for chain-extending dihydroxyl terminated pre-PLA. The resulting materials (PPLA) showed relevant flame-retardant properties and the use of only 5 wt.% PPLA into PLA enables obtaining a blend with good flame-retardant properties. Up to 10 wt.%, the so-obtained material presented Limited Oxygen Index of 35, a lower peak heat release rate (pHRR), a longer time to ignition, and V0 classification at the UL-94 test.

In this work, a three-step strategy aiming to prepare novel inherently flame-retardant polylactide-based materials is described. First, a bifunctional phosphorous-based diamine was prepared through the reaction between 9,10-dihydro-oxa-10-phosphaphenanthrene-10-oxide (DOPO) and 4,4′-diaminobenzophenone (DABP). This bifunctional diamine was thus used as an initiator for bulk ring-opening polymerization (ROP) of L,L-lactide (L,L-LA). The last step consisted of the bulk chain-coupling reaction of the so-obtained phosphorylated PLA oligomers with hexamethylene diisocyanate (HDI). DOPO was selected for this application because of its flame-retardant action, which is effective at a low P content (1 to 2 wt.%). In fact, DOPO presents an interesting flame-retardant effect because it acts in both condensed and gas phases [19]. During pyrolysis, DOPO-based flame retardants release reactive species in the gas phase (most probably PO°) that are very effective to achieve efficient flame inhibition, while the phosphorous remaining in the condensed phase promotes the formation of char residue.

The structure of the obtained phosphorylated PLA oligomers (DOPO-PLA) as well as the corresponding chain extended material (DOPO-PLA-PU) were characterized using Proton and Phosphorus nuclear magnetic resonance (1H & 31P NMR respectively) as well as by size exclusion chromatography (SEC), while their thermal properties and flame-retardant properties were evaluated by differential scanning calorimetry (DSC), thermal gravimetrical analysis (TGA), UL-94, and cone calorimeter tests, respectively. The obtained results evidenced superior flame-retardant properties of the novel phosphorylated PLA when tested alone and in blend with commercial PLA.

## 2. Materials and Methods

### 2.1. Materials

The L,L-LA (Purasorb® L, optical purity > 99.5%, MW = 144 g·mol^−1^, free acid < 1 meq·kg^−1^, water content < 0.02%, Purac Biochem BV, The Netherlands) and hexamethylene diisocyanate (HDI, purris. ≥99.0%, MW = 168 g·mol^−1^, Sigma-Aldrich, (Diegem, Belgium) were stored in a glove box prior to use. Tin(II) 2-ethylhexanoate (Sn(Oct)_2_, 95%, MW = 405 g·mol^−1^) and 4,4′-diaminobenzophenone (DABP, 97%, MW = 212 g·mol^−1^) from Sigma-Aldrich (now Merck) were used as received. 9,10-Dihydro-oxa-10-phosphaphenanthrene-10-oxide (DOPO, >97 %, MW = 216 g·mol^−1^, ADD APT Chemicals AG, The Netherlands) was recrystallized from tetrahydrofurane (THF) prior to use. All solvents (that is, methanol, chloroform, and THF; VWR, Belgium) were of analytical grade and used as received. Polylactide (Ingeo™ Biopolymer 3052D) from NatureWorks (Minnetonka, MN, USA) was used in this study as a commercial PLA resin. PLA 3052D is a semi-crystalline polymer with an average molecular weight Mn of 90,000 g/mol.

### 2.2. Syntheses

DOPO-diamine

The DOPO-diamine was obtained according to the literature [20]. Briefly, 32 g (0.15 mol, 1 eq) of DOPO and 5.35 g (0.25 mol, 1.7 eq) of DABP were placed into a 250 ml round-bottom glass-flask and heated at 180 °C for 3 h. The obtained homogeneous mixture was then allowed to cool-down to 100 °C, followed by the addition of 150 mL of toluene in order to precipitate the DOPO-diamine. The product was filtered and recrystallized repeatedly (×3) from THF (150 mL). Yield: 55%. 1H NMR (500 MHz, DMSO-d6, δ ppm): 8.5–6.5 (m, 20H, CHphenyl), 5.9 (br.dt, 4H, CHphenyl-NH2), 4.9 (br.s, 4H, NH2). 31P NMR (500 MHz, DMSO-d6, δ ppm): 31.78, 30.12. DSC (H/C/H, 10 °C·min^−1^, 0–200 °C): mp. 325 °C.

DOPO-PLA

DOPO-PLA was prepared via DOPO-diamine initiated ROP of L,L-LA in bulk following the procedure described hereafter: ROP of L, L-LA as initiated by DOPO diamine was performed in specially designed 250 mL Inox™ reactors (Maximator France, Rantigny, France) at 180 °C and a stirring rate of 50 rpm for 90 min. Slight nitrogen pressure (0.4 bar) was introduced in order to ensure an inert atmosphere and a premixing time of 10 min was used. Then, 50 g of L,L-LA (347 mmol) was mixed with 22 g of DOPO-diamine (35 mmol) and 1.4 g (3 mmol) of Sn(Oct)_2_. The molar ratio [L,L-LA]0/[DOPO-diamine]0 was fixed to 10. Stannous (II) octoate was used as catalyst at molar ratio [L,L-LA]0/[Sn(Oct)_2_]0 = 100. 

The as-obtained DOPO-PLA was recovered by solubilization in THF and precipitation in cold methanol.

DOPO-PLA PUs

DOPO-PLA PU was prepared via reactive extrusion using a 15 cm^3^ vertical corotational twin-screw DSM microcompounder (DSM, Sittard, The Netherlands), equipped with nitrogen inlet and water-cooling systems at 160 °C. Typically, 14 g of DOPO-PLA (5 mmol) was introduced into the extruder at 30 rpm and allowed to melt for 3 min, after which 0.98 mL HDI (5.8 mmol) was added and the reaction was allowed to continue at 30 rpm for 90 min.

Blending

Blending of DOPO-PLA-PU with commercial PLA was carried out in a Brabender internal mixer at 180 °C for 10 min (2 min mixing at 30 rpm followed by 8 min at 60 rpm). For the mass loss calorimeter test, plates (100 × 100 × 3 mm^3^) were compression molded at 180 °C using an Agila PE20 hydraulic press. More precisely, the material was first pressed at a low pressure for 200 s (three degassing cycles), followed by a high-pressure cycle at 150 bars for 180 s. The samples were then cooled down under pressure (80 bars).

### 2.3. Methods

1H and 31P NMR spectra were collected with a Bruker AMX-500 at a frequency of 500 MHz instruments, respectively, in Hexadeuterodimethyl sulfoxide (DMSO-d6).

Size exclusion chromatography (SEC) was used to determine the molar mass of the prepared materials. Analyses were performed at 30 °C in chloroform (CHCl_3_) using an Agilent liquid chromatograph equipped with an Agilent degasser, an isocratic High-performance liquid chromatography (HPLC) pump (flow rate = 1 mL·min^−1^), an Agilent autosampler (loop volume = 200 µL, solution conc. = 2.5 mg·mL^−1^), an Agilent-DRI refractive index detector, and three columns: a PL gel 10 µm guard column and two PL gel Mixed-D 10 μm columns (linear columns for separation of MWPS ranging from 500 to 106 g·mol^−1^). Polystyrene standards were used for calibration.

Thermal gravimetrical analyses (TGAs) were performed with a TA Q500 thermogravimetric analyzer (Zellik, Belgium). The weight loss was recorded upon heating the samples (having initial masses of ca. 10 mg) at 20 °C·min^−1^ from 30 to 700 °C, under N_2_ flow of 60 mL·min^−1^. The referred temperatures of maximum degradation rate (Tmax) were taken as the inflection point of the sigmoidal steps (i.e., the maxima of the first-derivative curve).

Differential scanning calorimetry (DSC) measurements were performed with a TA Q200 calorimeter (Zellik, Belgium) calibrated with high purity indium and operating under N_2_ flow. The sample weight was about 5 mg and a scanning rate of 10 °C·min^−1^ was employed in all the runs. Each run followed a heat/cool/heat (H/C/H) procedure from 0 to 200 °C. The reported thermal transition values, Tg (glass transition temperature) and Tm (melting temperature), were defined as the midpoints of the sigmoidal curve, minima of the exotherms, and maxima of the endotherms, respectively.

The fire behavior of the DOPO-PLA-PU was evaluated using the mass loss cone calorimeter test at 35 kW·m^−2^. Cone calorimeter is one of the most used devices to assess the flammability of materials at bench scale. The peak of heat release rate (pHRR), time to ignition (TTI), as well as total heat release (THR), which are considered as the most important parameters in this fire test, were considered. A high pHRR and a low TTI may cause both fast ignition and rapid-fire propagation. Mass loss cone calorimeter tests were performed according to ISO 13927 standard procedures with a Fire Testing Technology Limited (FTT) mass loss cone calorimeter. Samples (100 × 100 × 3 mm^3^) were exposed to an external heat flux of 35 kW·m^−2^, corresponding to common heat flux in a mild fire scenario. UL-94 test was performed on films of 0.8 mm thickness.

## 3. Results

### 3.1. DOPO-Diamine Initiator Preparation and Characterizations

**Scheme 1:**
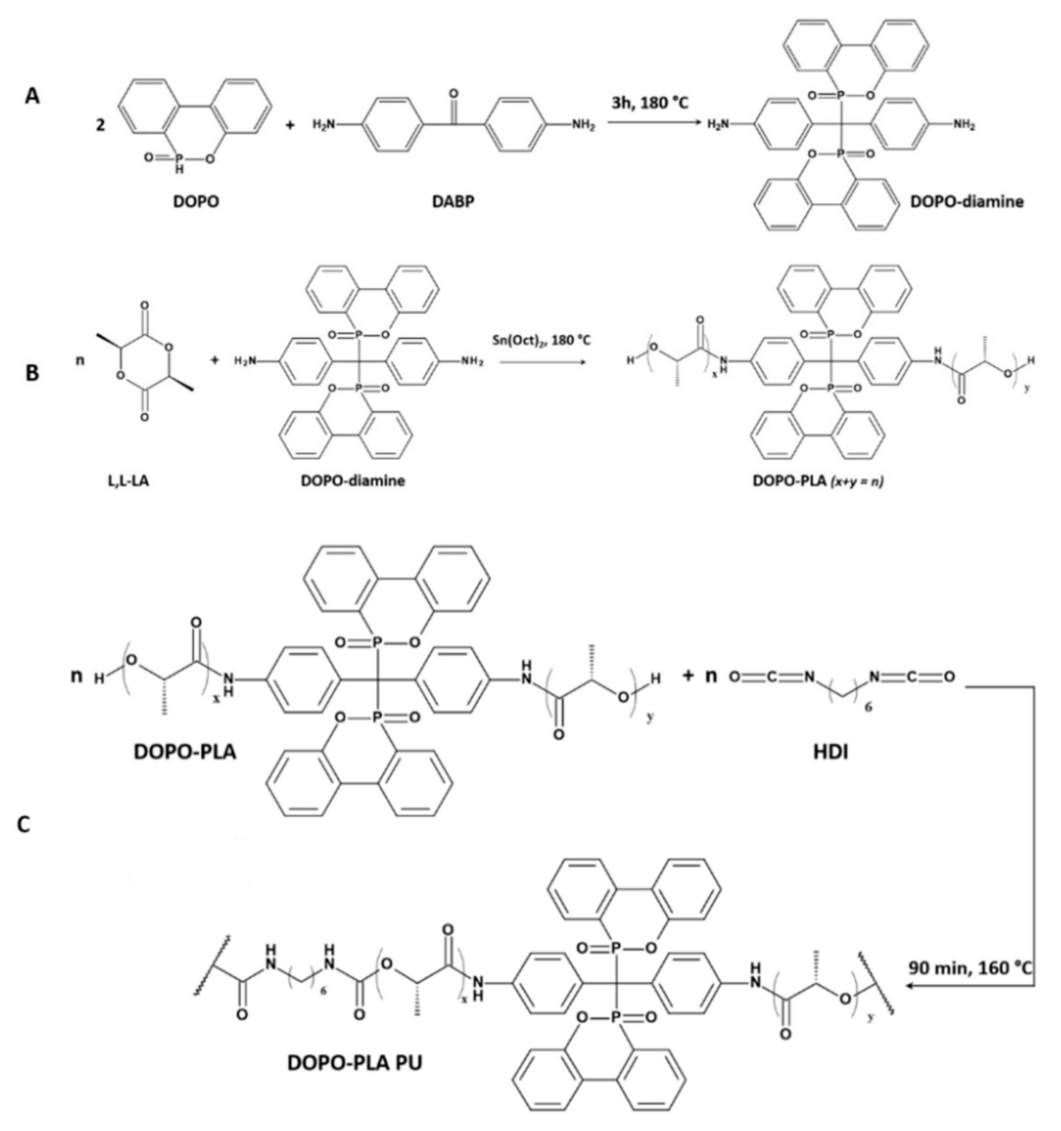
Three-step synthetic pathway to 9,10-dihydro-oxa-10-phosphaphenanthrene-10-oxide (DOPO)-polylactide (PLA) PUs: (**A**) Synthesis of DOPO-diamine; (**B**) DOPO-initiated bulk ring-opening polymerization (ROP) of L,L-lactide (L,L-LA); (**C**) chain coupling reaction.

DOPO-diamine was synthesized through a well-established procedure (Scheme 1A) starting from 9,10-dihydro-oxa-10-phosphaphenanthrene-10-oxide (DOPO) and 4,4’-diaminobenzophenone (DABP). [20] Global yield reached 55% after recovery of the product obtained after three hours at 180 °C. In accordance to the state-of-the-art, 1H and 31P NMR characterizations concluded on the formation of the expected DOPO-diamine.

**Figure 1:**
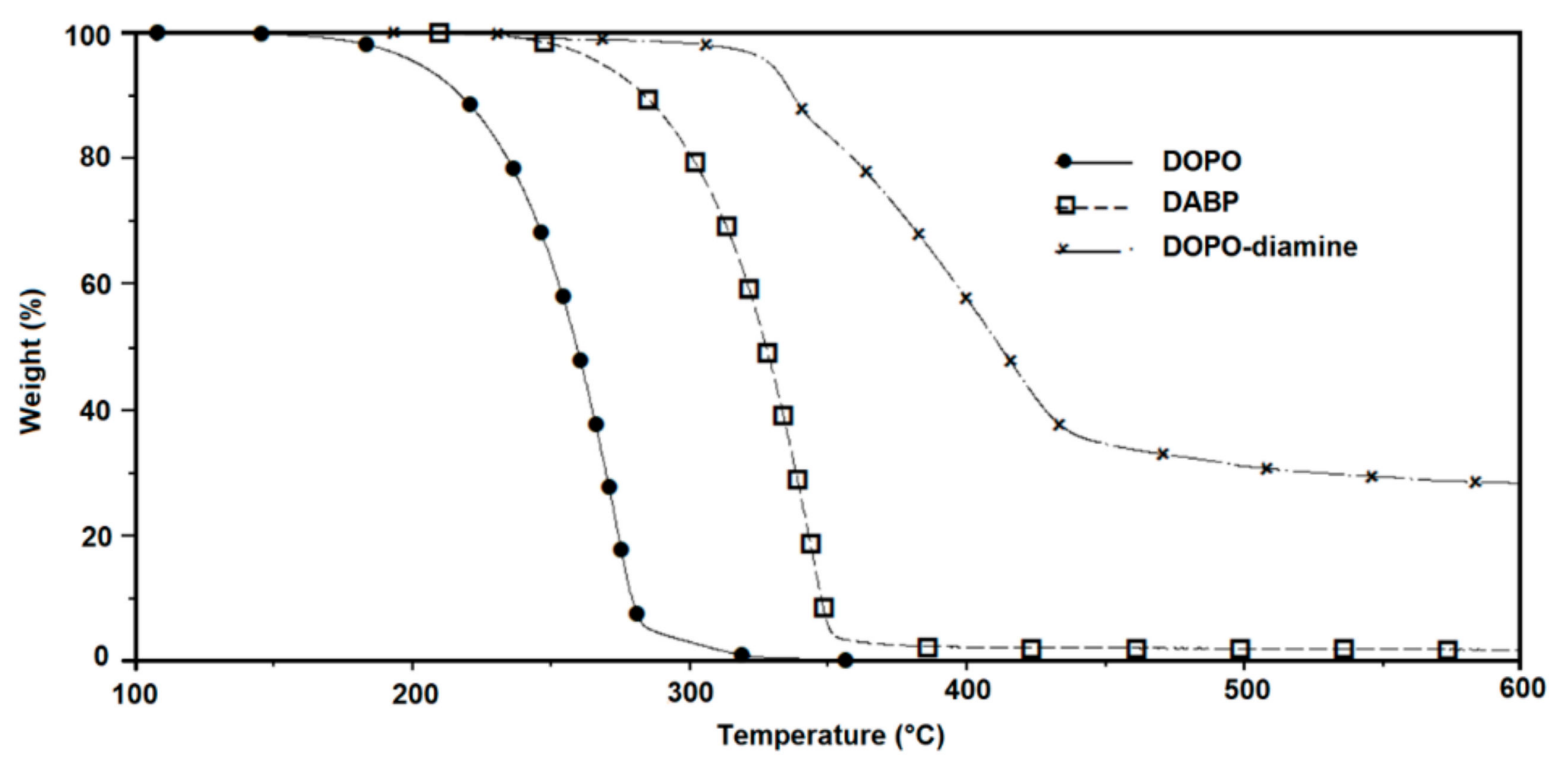
Thermogravimetric analyses (TGAs) performed on 9,10-dihydro-oxa-10-phosphaphenanthrene-10-oxide (DOPO), DABP, and DOPO-diamine at 20 °C/min under nitrogen.

Prior to its use as a polymerization initiator, the thermal stability of the DOPO-diamine was studied by thermogravimetric analysis (TGA) and compared to both DOPO and DABP reactants (Figure 1). Clearly, the DOPO-diamine is characterized by a well-higher thermal stability as compared with its precursors. With a thermal degradation starting above 300 °C, the DOPO-diamine TGA analysis also reveals the formation of a char residue of 27 wt.% at circa 450 °C. Those results are of prime importance and indicate that DOPO-diamine could be processed at PLA melt processing (180 °C) without undergoing any thermal degradation.

### 3.2. Synthesis and Properties of DOPO-PLA Oligomers

The DOPO-diamine was then employed as a telechelic initiator for the ring-opening polymerization (ROP) of L,L-LA in the presence of tin(II) octanoate (Sn(Oct)_2_). Duda et al. showed that the mechanism of LA ROP from primary amine and catalyzed by Sn(Oct)_2_ does not differ from an ROP process initiated from an alcohol exogenous initiator [21]. In the specific case of the DOPO-diamine, a controlled “coordination-insertion” ROP of L,L-LA would proceed from both primary amines, leading to a DOPO core functionalized by two PLA segments end-capped by hydroxyl groups (Scheme 1B). To get rid of the inherent insolubility of the DOP-diamine in most of the studied organic solvents, the L,L-LA ROP process was performed in bulk at 180 °C for an initial monomer-to-initiator ratio ([L,L-LA]0/[DOPO-diamine]0) of 10. Note here that such a ratio was selected to produce an oligo-PLA composed by a sufficiently high atomic percentage in phosphorus atoms (4 wt.%) to impact the fire properties. To ensure a controlled process, a [DOPO-diamine]0/[Sn(Oct)_2_]0 molar ratio of 12/1 was used [22]. After 90 min of reaction, a 1H NMR analysis of the crude medium revealed a conversion in L,L-LA of 96%. By comparing the relative intensities of free DOPO-diamine to the one incorporated into the DOPO-PLA, an efficacy of initiation as high as 70 mol% was calculated. After precipitation of the sample, and considering that the DOPO-diamine initiated the L,L-LA ROP symmetrically, an experimental molar mass (Mn, NMR) of 2100 g/mol was calculated by 1H NMR spectroscopy, which is in good agreement with the theoretical value (Mn, th = 2000 g/mol).

**Figure 2:**
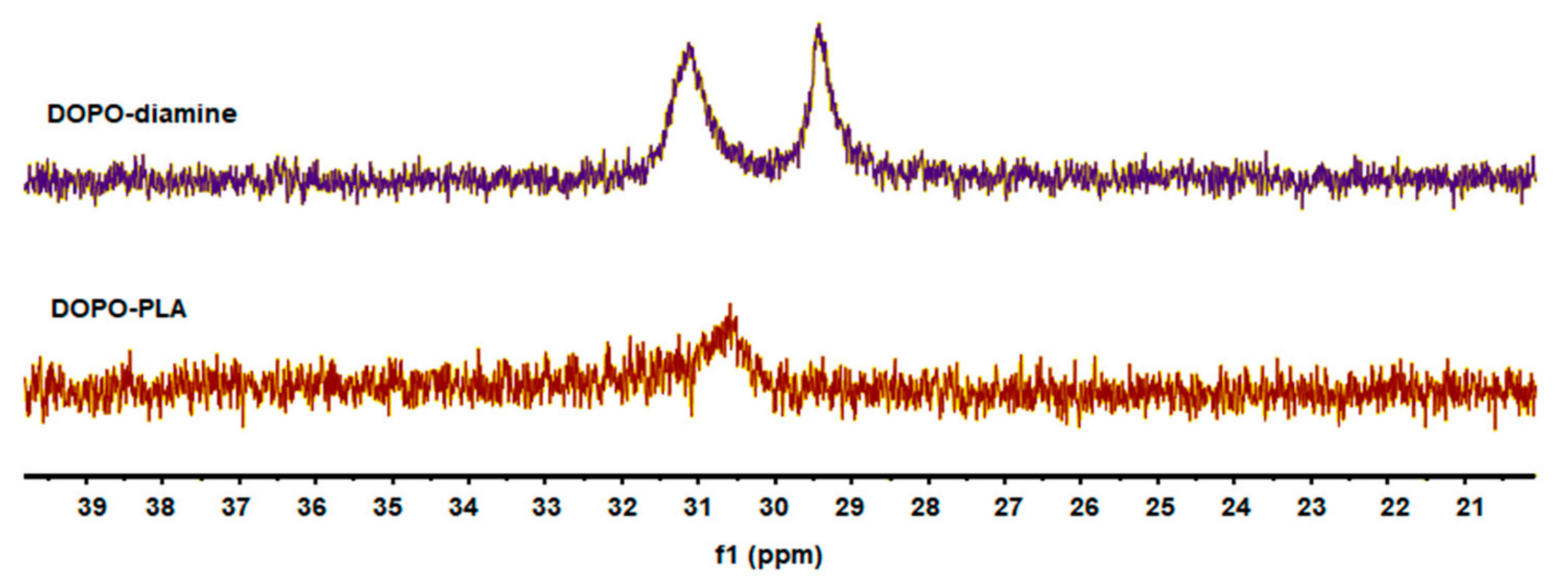
^31^P NMR analyses (zoomed between 40 and 20 ppm) of DOPO-diamine and DOPO-polylactide (PLA)-PU.

In comparison with the two phosphorus signals observed by analysis of the pristine DOPO-diamine before polymerization (at 29 and 31 ppm), the 31P NMR analysis of the as-obtained DOPO-PLA confirmed the incorporation of the DOPO initiator into the PLA chain by the presence of one single phosphorus peak at 30.5 ppm (Figure 2) [20].

**Figure 3:**
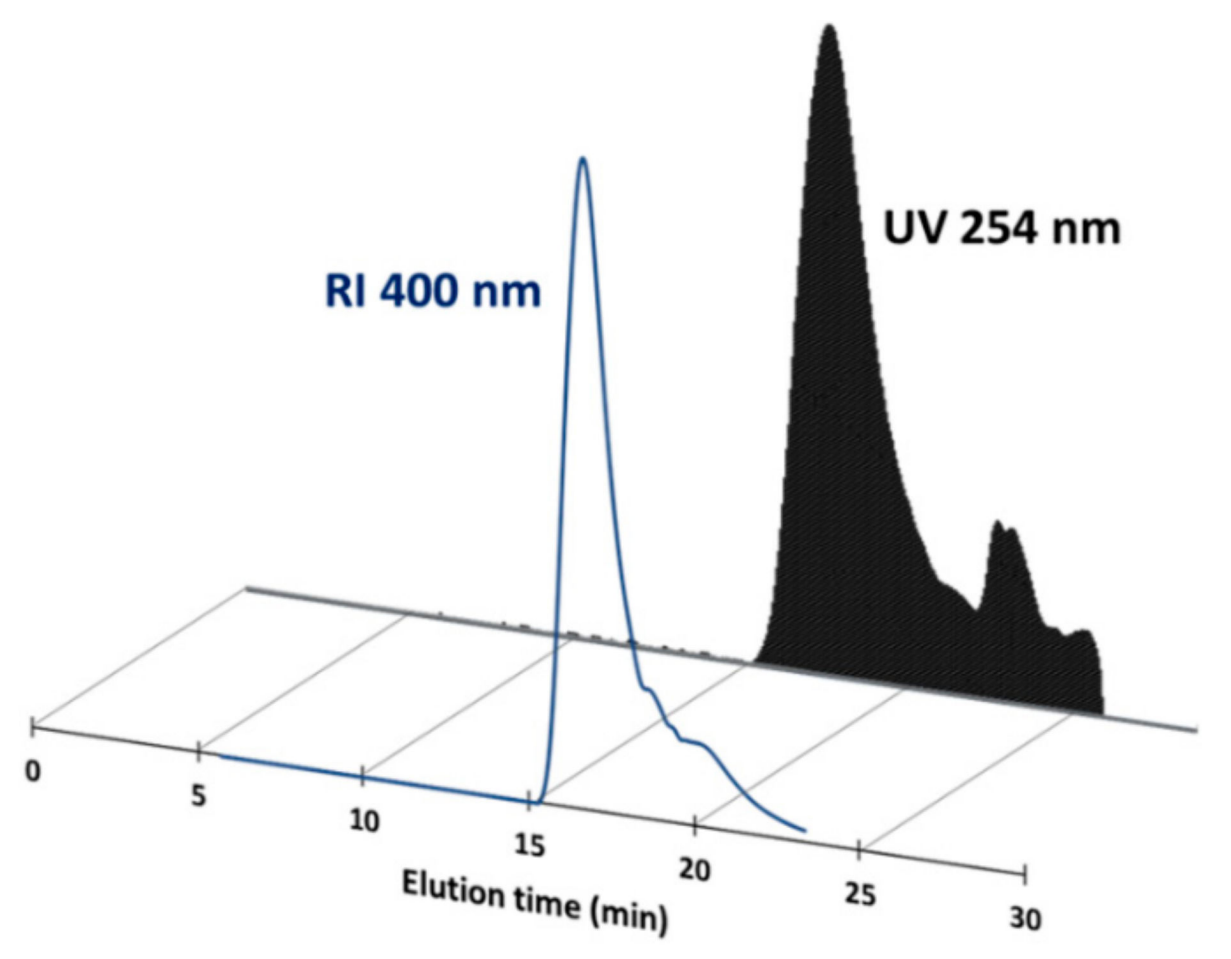
Size exclusion chromatography (SEC) elution curves of DOPO-PLA using both RI and UV detectors.

Size exclusion chromatography (SEC) traces of the polylactide generated from the DOPO-diamine initiator (Mn = 2700 g·mol^−1^, Ð = 1.5) and using both refractive index and UV detectors (254 nm, respectively) clearly show that DOPO is distributed throughout the sample (Figure 3). Indeed, SEC in dual RI and UV detection is a well-known method providing information on the composition at any point in the molar mass distribution when at least one component absorbs at a suitable UV-wavelength [23]. With this respect and knowing that DOPO is the only element in DOPO-PLA that absorbs at a UV-wavelength, it was possible to confirm its presence at any point of the molar mass distribution by the UV elution curve precisely following the RI one, as shown in Figure 3.

**Figure 4:**
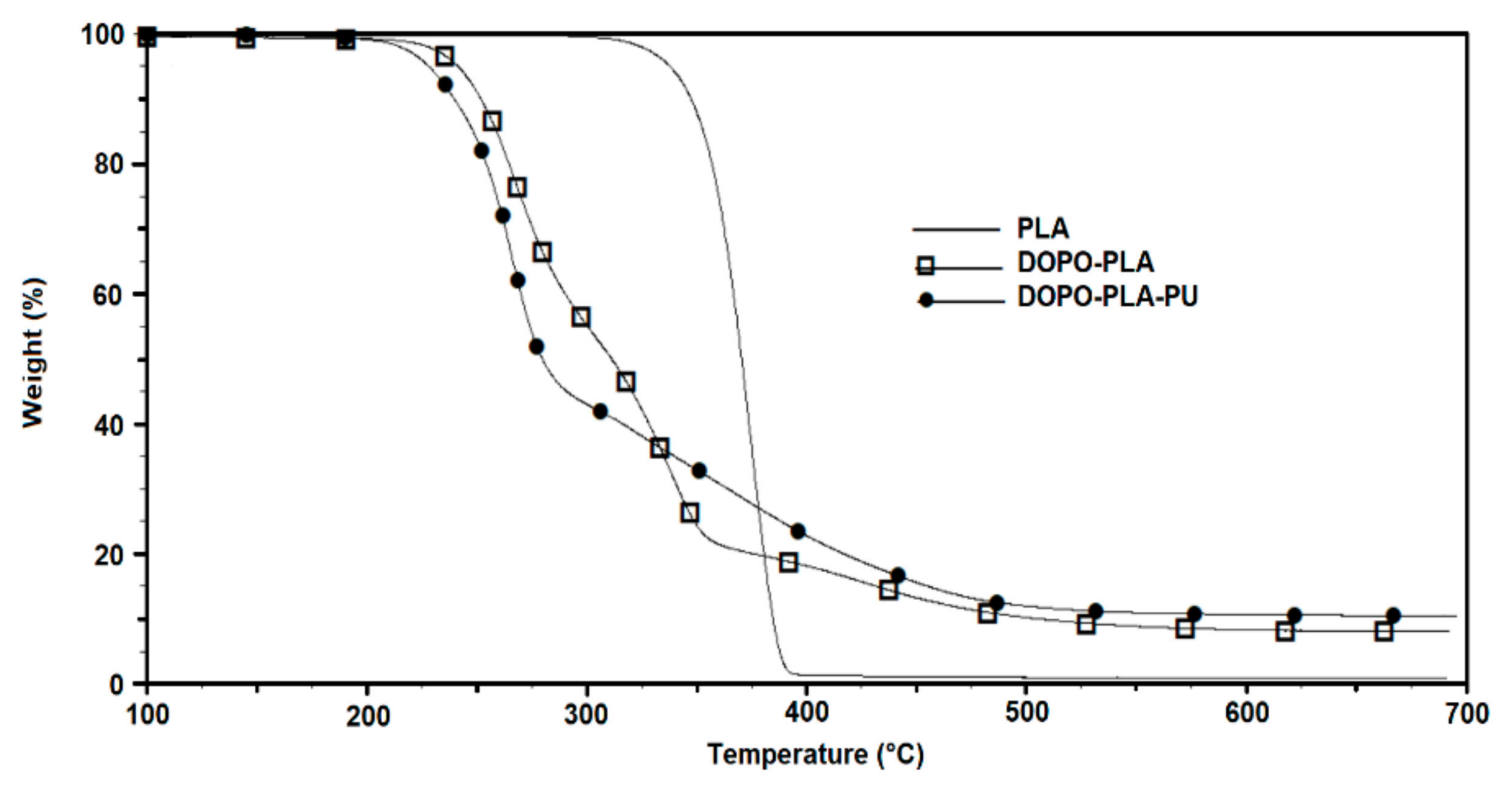
TGA curves of phosphorylated PLA oligomers (DOPO-PLA) and chain extended phosphorylated oligomers (DOPO-PLA-PU) in comparison with commercial PLA, 20 °C/min under nitrogen.

Finally, a TGA analysis was performed on the DOPO-PLA oligomer (Figure 4) and the results summarized in Table 1. In comparison with high commercial molecular weight PLA (see experimental section), DOPO-PLA oligomers show lower thermal stability because their decomposition starts at 241 °C (T_5%_), while the weight loss of commercial PLA occurs at 300 °C. The different thermal stability observed between these two materials is the result of their high difference in molecular weight. PLA thermal stability is highly dependent on Mn and low molecular weight chain starts to decompose at a lower temperature [24,25,26]. Interestingly, TGA curves also evidence another behavior. In fact, during their thermal decomposition, DOPO-PLA oligomers generate some char, while commercial PLA totally degrades during the analysis. This result highlights the ability of DOPO to act in the condensed phase by promoting the formation of char structures.

### 3.3. Synthesis and Properties of DOPO-PLA-PU by Chain Extension

**Figure 5:**
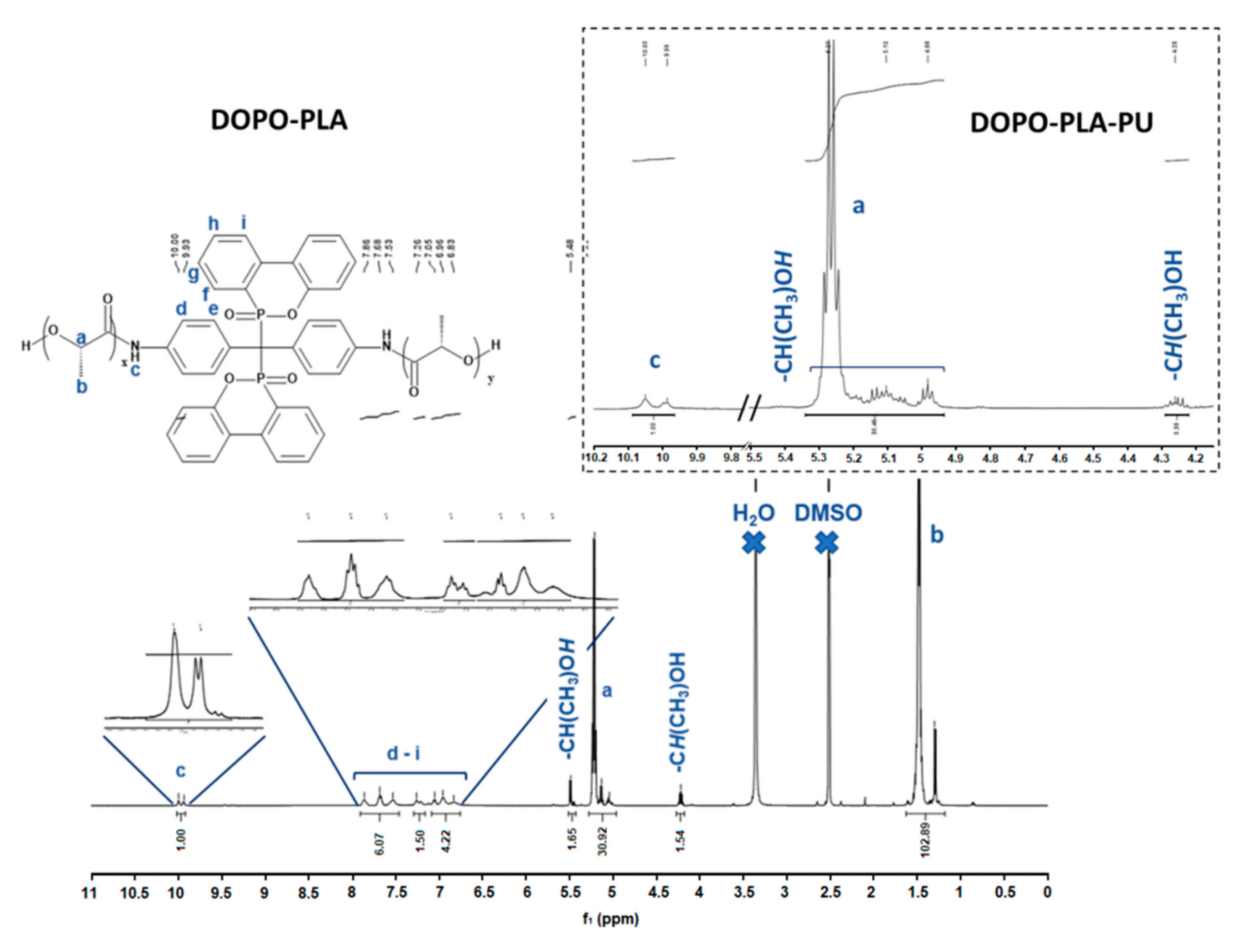
^1^H NMR spectrum of DOPO-PLA recorded in DMSO-d6. The figure inset shows the ^1^H NMR analysis of DOPO-PLA-PU (only the zoom between δ = 4.2 and 10.2 ppm is shown for clarity).

DOPO-PLA oligomers were thus further subjected to chain-extension reaction using HDI via reactive extrusion in order to increase their overall molecular weight. As evidenced by 1H NMR spectroscopy (Figure 5, inset), DOPO-PLA reacted well with the diisocyanate moiety. Indeed, the signal corresponding to the hydroxymethine-PLA end groups (–CH(CH_3_)OH), initially present at 4.25 ppm (Figure 5), almost completely disappeared, as well as the hydroxy proton end groups (-CH(CH3)OH) at 5.48 ppm, to the benefit of new urethane bonds. While integrations of amide protons (Hc, 10 ppm) and methine repeating units (Ha, 5.18 ppm) are logically identical before and after the chain extension experiment, the diminushion of the methine PLA end groups (4.25 ppm) from an integration of 1.54 to 0.39 allows an efficiency of reaction of 78% to be calculated. Note here that, owing to the limited PUs solubility in common solvents [27] and the possible interactions of the as-obtained DOPO-PLA-PU with the SEC columns, resulting in truncated Mn value, no SEC analysis was realized. It is worth mentioning that the incorporation of urethane functions in the backbone of PLA macromolecule does not affect its compostability [28].

**Figure 6:**
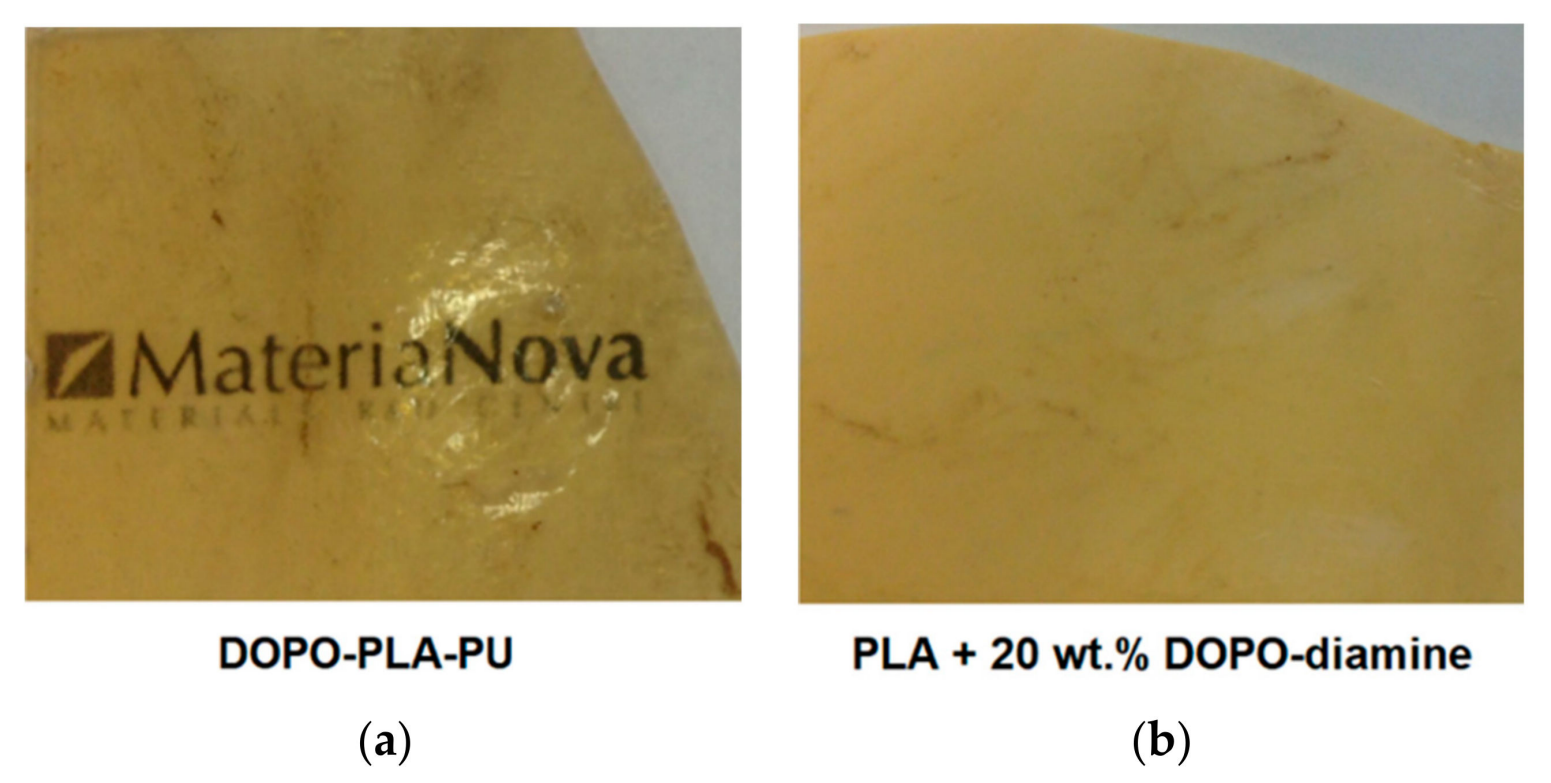
Films (thickness of ca. 0.8 mm) of transparent DOPO-PLA-PU (**a**) and the opaque blend of commercial PLA containing 20 wt.% of DOPO-diamine as additive (**b**).

**Figure 7:**
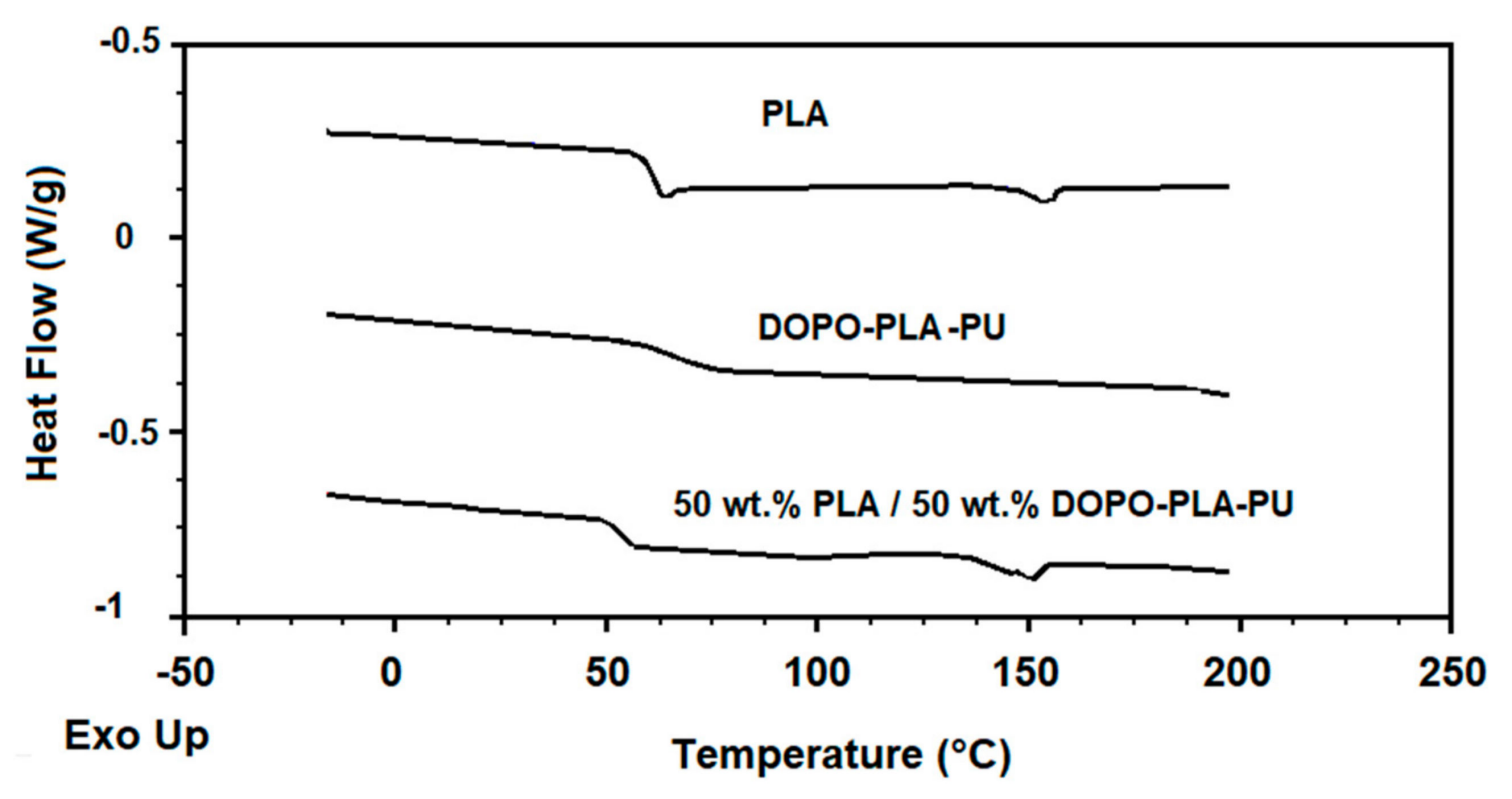
Differential scanning calorimetry (DSC) heating curves (second run) of commercially available PLA, DOPO-PLA-PU, and their blend 50/50 (wt/wt.%).

The resulting DOPO-PLA-PU produced is in the form of a transparent yellow material (Figure 6). The DSC results specify that it is an amorphous polymer characterized by a Tg of 60 °C, similar to that of unmodified PLA (Figure 7 and Table 2). The presence of DOPO-diamine and urethane functions hinders the crystallization of DOPO-PLA-PU polymer. However, the thermal stability of chain extended DOPO-PLA is slightly reduced in comparison with the corresponding oligomers. Further, the presence of urethane moieties also enables a slight increase in the amount of residue (+2%) (Figure 4 and Table 1).

**Figure 8:**
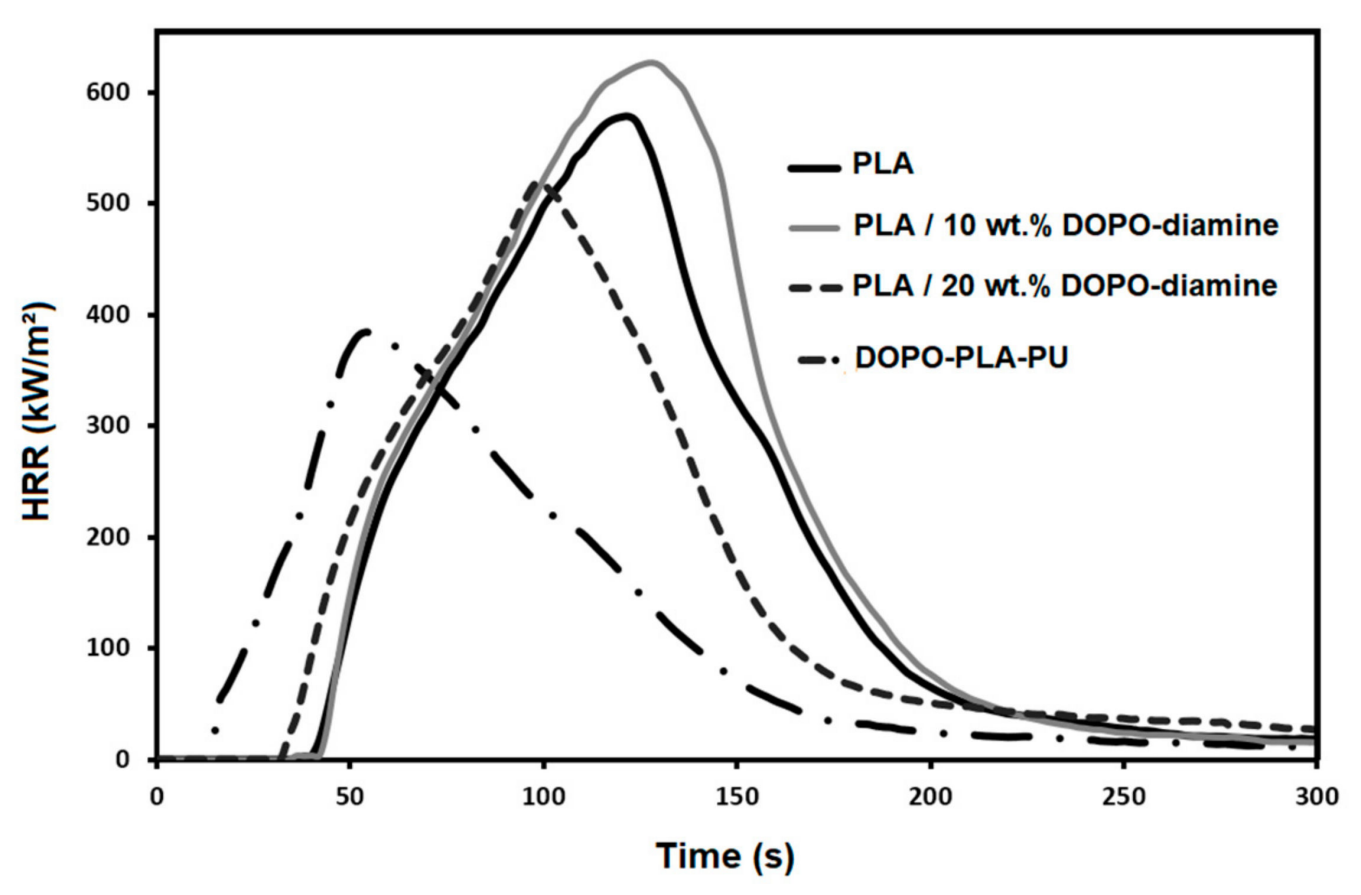
Heat release rate (HRR) curves obtained during the mass loss cone calorimeter test at 35 kW/m^2^.

**Figure 9:**
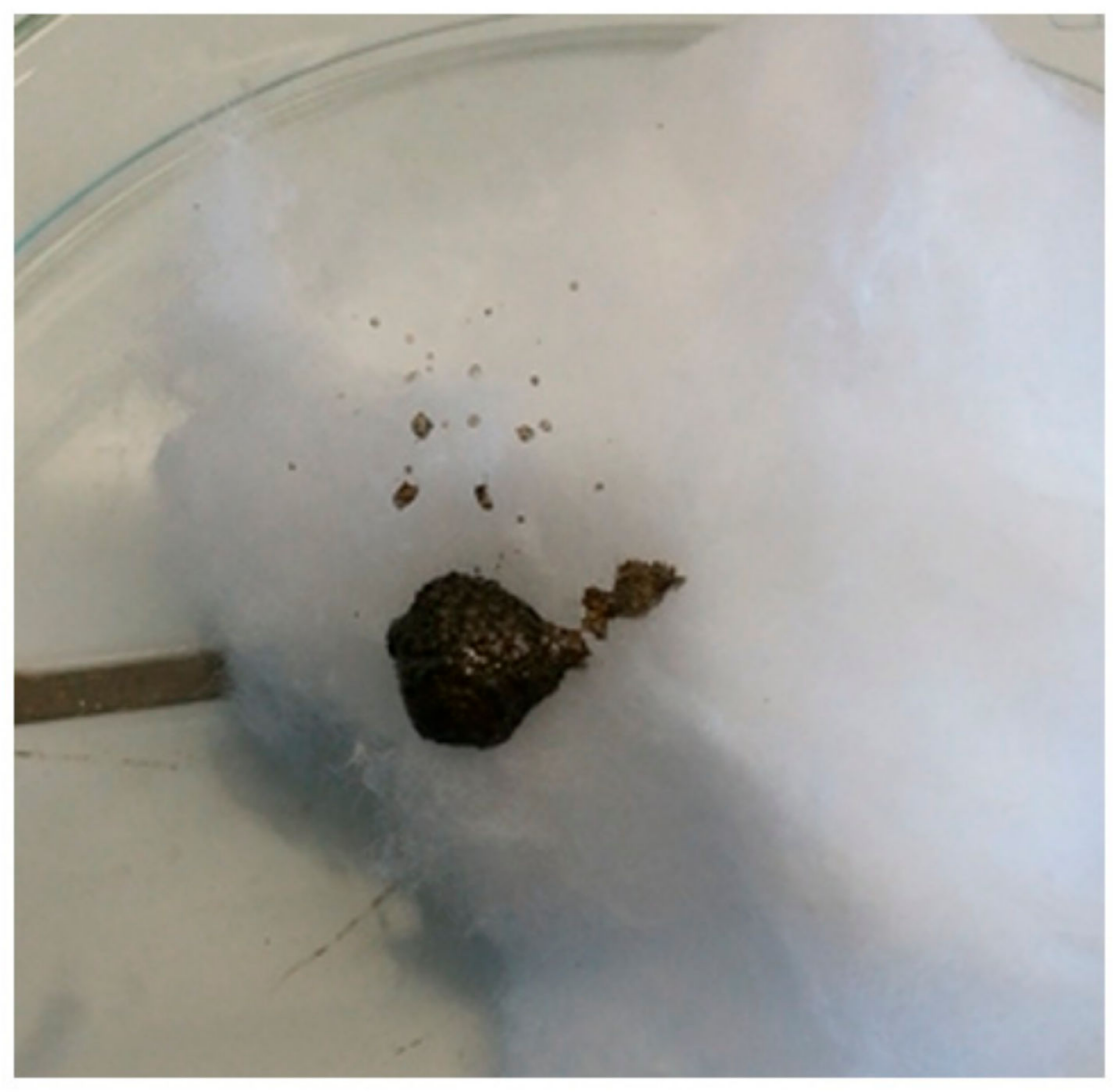
Photograph showing the formation of black residue at the surface of cotton after the UL-94 test.

The flame-retardant behavior of DOPO-PLA-PU was assessed using mass loss cone calorimeter. For comparison, Figure 8 and Table 3 summarize the results obtained during the combustion of DOPO-PLA-PU and the commercially available PLA. Pristine PLA exhibits a strong combustion, starting after 40 s, consuming all the material and releasing a total heat of about 54 MJ/m^2^ with a pHRR of 580 kW/m^2^. In the case of DOPO-PLA-PU, a lower pHRR (−35%) and THR (−36%), as well as a reduction of the time to ignition from 40 to 16 s, were observed. It is worth mentioning that, during the combustion of DOPO-PLA-PU, a char residue was formed at the surface of the burning material, evidencing the condensed phase action of the DOPO contained in the material. However, this char was not thermally stable and only a few pieces remain at the end of the test. The formation of the char during the combustion is responsible for the reduction of HRR, but it is not excluded that a gas phase may also occur. Indeed, DOPO and its derivatives act in both gas and condensed phases by the release of low molecular weight phosphorus based species that can scavenge the H° and OH° radicals in the flame and the generation of char layer. [29] The flame-retardant effect in the gas phase cannot be excluded as UL-94 tests performed on films of 0.8 mm thickness highlighted that the material was very difficult to ignite. A flame inhibition owing to the generation of active P-based species seems to be responsible for this resistance to ignition in addition to the char formation. In fact, during the UL-94 test, the material is very difficult to ignite, and melts, but drops do not induce any cotton ignition and only a black residue is formed at the surface of cotton (Figure 9). The low time to ignition observed in the case of DOPO-PLA-PU is the result of the low thermal stability of this polymer that starts to decompose around 100 °C, thus earlier than the comparative commercial PLA (Figure 4). The reduced thermal stability of DOPO-PLA-PU is likely the result of the lower molecular weight of DOPO-PLA-PU with respect to the commercial PLA. In fact, Figure 4 showed that the TGA curve of DOPO-PLA-PU is close to that of the starting phosphorylated PLA oligomers, meaning the urethane functions do not affect the material thermal stability.

The high weight phosphorus content in DOPO-PLA-PU (4%) is thus beneficial and allows obtaining a material presenting good flame-retardant performances. However, an in-depth study is needed for better establishing the real contribution of DOPO when combined with DABP and integrated in the backbone of PLA macromolecules.

Interestingly enough, when DOPO-diamine is physically added to PLA at 10 wt.% and 20 wt.% content, the so-obtained material does not present any flame-retardant behavior during the cone calorimeter test (Figure 8 and Table 3). Moreover, using the additive route also affects the transparency of the material, which becomes opaque (Figure 6b).

**Figure 10:**
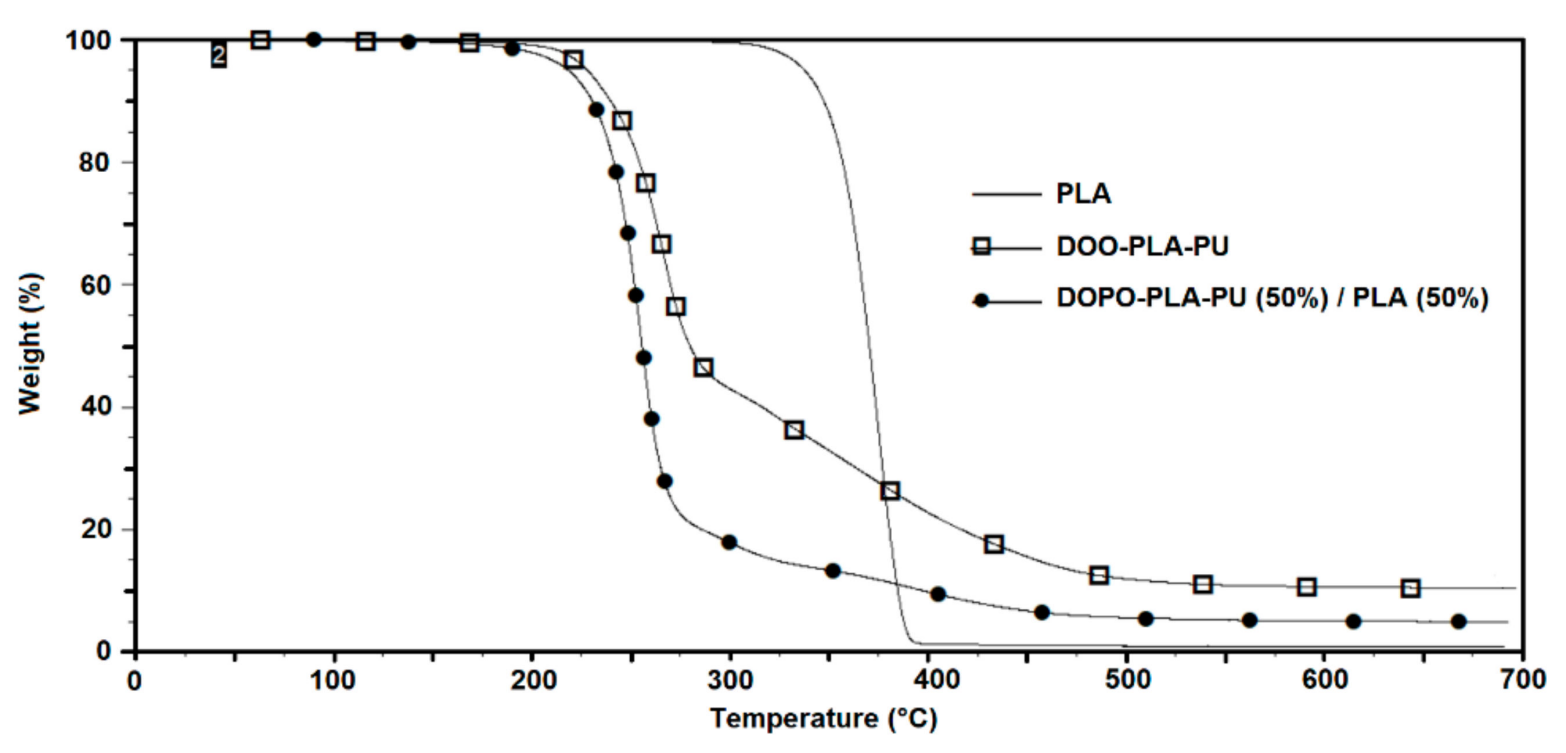
TGA curves under nitrogen (20 °C/min).

Melt blending DOPO-PLA-PU with commercial PLA presents another interesting way for taking advantage of the good flame-retardant properties of DOPO-PLA-PU. With this view, a new blend was prepared by melt blending the two polymers in an internal mixer. TGA analysis (Figure 10) shows that the incorporation of 50 wt.% DOPO-PLA-PU induces an important reduction of PLA thermal stability, which becomes similar to that of the phosphorylated PLA-PU, but unfortunately does not promote the formation of further residue, as all PLAs continue to decompose and only a final residue corresponding to that generated by the thermal degradation of DOPO-PLA-PU is obtained. Interestingly, DSC analyses show that the presence of DOPO-PLA-PU also induces a reduction of Tg of the blend that decreases from 60 °C for both polymers separately to 50 °C when combined. This result evidences the good miscibility between both polymers where DOPO-PLA-PU acts as plasticizer for the PLA phase. Moreover, the so-obtained material enables obtaining transparent films.

Blending DOPO-PLA-PU (50 wt.%) with commercial PLA presents a good strategy because the UL-94 test, performed on 0.8 mm thick films, allows for reaching a remarkable V0 classification, while commercial PLA simply burns after the first flame application, forming burning drops that induce cotton inflammation. This strategy could be also applied for PMMA, as PLA and PMMA demonstrate good miscibility [30] and blending DOPO-PLA-PU with pristine PMMA could enable enhancing PMMA flame-retardant properties, while maintaining the material transparency.

## 4. Conclusions

Organophosphorus flame-retardant, a bifunctional DOPO-diamine, was successfully prepared and chemically incorporated into the PLA backbone by combining ring-opening polymerization and reactive extrusion. The bifunctional amine was first prepared by reacting DOPO with 4,4’-diaminobenzophenone and used as an initiator for bulk ring-opening polymerization of L,L-lactide (L,L-LA). The last step consisted of the bulk chain-coupling reaction of the so-obtained phosphorylated PLA oligomers with hexamethylene diisocyanate (HDI) by reactive extrusion.

Chemical structures of the phosphorylated PLA oligomers and its chain extended homologue were confirmed by 1H, 31P NMR, and SEC analyses. The thermal analysis revealed that DOPO-PLA oligomers presented lower thermal stability than commercially available PLA, but superior flame-retardant properties. Cone calorimeter tests evidenced a pHRR and THR reduction of about 35 and 36%, respectively, for DOPO-PLA-PU with respect to commercially available PLA. Moreover, DOPO-PLA-PU reached V0 classification at the UL-94 test. Combining DOPO-PLA-PU with PLA was shown to present an interesting way for developing transparent PLA films (0.8 mm thickness) with V0 classification.

All these results demonstrated that this novel synthesis route enables the development of inherent flame-retardant PLA. Ongoing works are underway in our laboratory to understand the mode of action of DOPO-diamine when incorporated into PLA backbone.

## Figures and Tables

**Table 1:** Characteristic degradation temperatures and residues amounts from thermogravimetric analysis (TGA) experiments (under N_2_ and at 20 °C/min) for commercial polylactide (PLA), phosphorylated PLA oligomers (9,10-dihydro-oxa-10-phosphaphenanthrene-10-oxide (DOPO)-PLA), chain extended phosphorylated PLA oligomers (DOPO-PLA-PU), as well as PLA/DOPO-PLA-PU blend.

Sample	T_5%_ (°C)	T_max_ (°C)	Residue at 700 °C (%)
PLA	337	374	0.4
DOPO-PLA	241	267; 338	8.5
DOPO-PLA-PU	228	265	10.4
50% DOPO-PLA-PU/50% PLA	218	255	5

**Table 2:** Glass transition (Tg), melting temperature (Tm), and melting enthalpies (∆Hm) obtained by differential scanning calorimetry (DSC) second heating run.

Sample	T_g_ (°C)	T_m_ (°C)	∆H_m_ (J/g)
PLA	60	154	2
DOPO-PLA-PU	60.5	–	–
50% DOPO-PLA-PU/50% PLA	50.5	150	3

**Table 3:** Cone calorimeter (35 kW/m^2^) and UL-94 results. pHRR, peak heat release rate; TTI, time to ignition; THR, total heat release.

Sample	TTI (s)	pHRR (kW/m^2^)	pHRR Reduction (%)	THR (MJ/m^2^)	THR Reduction (%)	UL-94 Classification
PLA	40	580	–	54	–	No rating
PLA/10 wt.% DOPO-diamine	42	620	No reduction	61.7	No reduction	No rating
PLA/20 wt.% DOPO-diamine	34	520	No reduction	51	No reduction	No rating
DOPO-PLA-PU	16	380	35	34.5	–36	V0
